# COVID-19 in patients with anemia and haematological malignancies: risk factors, clinical guidelines, and emerging therapeutic approaches

**DOI:** 10.1186/s12964-023-01316-9

**Published:** 2024-02-15

**Authors:** Sareh Kakavandi, Bahareh Hajikhani, Paniz Azizi, Fatemeh Aziziyan, Mohsen Nabi-Afjadi, Marzieh Ramezani Farani, Hamidreza Zalpoor, Maryam Azarian, Mahdiyar Iravani Saadi, Behrouz Gharesi-Fard, Evangelos Terpos, Iman Zare, Mohammad Motamedifar

**Affiliations:** 1 Department of Bacteriology and Virology, School of Medicine, Shiraz University of Medical Sciences, Shiraz, Iran; 2 Department of Microbiology, School of Medicine, Shahid Beheshti University of Medical Sciences, Tehran, Iran; 3 Psychological and Brain Science Departments, Program in Neuroscience, Indiana University, Bloomington, IN USA; 4 Department of Biochemistry, Faculty of Biological Sciences, Tarbiat Modares University, Tehran, Iran; 5 Department of Biological Sciences and Bioengineering, Nano Bio High-Tech Materials Research Center, Inha University, Incheon, 22212 Republic of Korea; 6 Student Research Committee, Fasa University of Medical Sciences, Fasa, Iran; 7 Network of Immunity in Infection, Malignancy & Autoimmunity (NIIMA), Universal Scientific Education & Research Network (USERN), Tehran, Iran; 8 Department of Radiology, Charité - Universitätsmedizin Berlin, 10117 Berlin, Germany; 9 Hematology Research Center, Shiraz University of Medical Sciences, Shiraz, Iran; 10 Department of Immunology, Shiraz University of Medical Sciences, Shiraz, Iran; 11 Department of Clinical Therapeutics, School of Medicine, National and Kapodistrian University of Athens, Athens, Greece; 12 Research and Development Department, Sina Medical Biochemistry Technologies Co., Ltd., Shiraz, 7178795844 Iran; 13 Shiraz HIV/AIDS Research Center, Institute of Health, Shiraz University of Medical Sciences, Shiraz, Iran

**Keywords:** COVID-19, SARS-CoV-2, Anemia, Haematological disorders and malignancies, Immune response

## Abstract

Extensive research in countries with high sociodemographic indices (SDIs) to date has shown that coronavirus disease 2019 (COVID-19) may be directly associated with more severe outcomes among patients living with haematological disorders and malignancies (HDMs). Because individuals with moderate to severe immunodeficiency are likely to undergo persistent infections, shed virus particles for prolonged periods, and lack an inflammatory or abortive phase, this represents an overall risk of morbidity and mortality from COVID-19. In cases suffering from HDMs, further investigation is needed to achieve a better understanding of triviruses and a group of related variants in patients with anemia and HDMs, as well as their treatment through vaccines, drugs, and other methods. Against this background, the present study aimed to delineate the relationship between HDMs and the novel COVID-19, severe acute respiratory syndrome coronavirus 2 (SARS-CoV-2). Besides, effective treatment options for HDM cases were further explored to address this epidemic and its variants. Therefore, learning about how COVID-19 manifests in these patients, along with exploiting the most appropriate treatments, may lead to the development of treatment and care strategies by clinicians and researchers to help patients recover faster.

Video Abstract

**Supplementary Information:**

The online version contains supplementary material available at 10.1186/s12964-023-01316-9.

## Introduction

As of December 2019, the first confirmed cases of new COVID-19, SARS-CoV-2, were reported in Wuhan, China, and the situation has affected more than 200 countries [1]. The infection may cause severe illness with shortness of breath and some symptoms of chest pain, which can potentially develop into pneumonia [2]. On the other hand, at the same time as the spread of this virus worldwide, its detection of this virus through laboratory tests such as real-time reverse transcription polymerase chain reaction (rRT-PCR), chest CT scans, Immunoglobulin Rapid Diagnostic Tests (Ig-RDTs), Elisa-linked Immunosorbent Assays (ELISAs), and detecting seroconverted IgA, IgM and IgG antibodies were performed in serum or blood [3, 4].

Therefore, the respiratory system involved during SARS-CoV-2 could be associated with the dysregulated expression of some biomarkers [5]. In addition, lymphopenia is nothing special in this condition and high patients, which indicates anemia in cases of COVID-19. The ferritin test shows higher than normal levels, indicating an acute inflammatory response in patients or the entry of the virus and its effects on iron metabolism, which can reduce the bioavailability of iron, thereby depriving the virus of this element and leading to anemia [6, 7]. As of October 2021, there have been abundant reports of high mortality rates in HDM patients co-infected with COVID-19 [8]. Notably, HDMs caused by overproduction of blood cells are assumed as an abnormal phenomenon that leads to improper control of these cells in fighting infections or preventing serious bleeding [9, 10]. Such malignancies and their treatment may affect the human immune system and put them at high risk of contracting COVID-19 and suffering its consequences [11]. As evidenced in related studies, patients with HDM, above all those with acute lymphoblastic leukemia (ALL), essential thrombocytopenia (ET), multiple myeloma (MM), and chronic myeloid leukemia (CML), compared to people without such conditions, have experienced a higher chance of infection with COVID-19 [12]. Like other ribonucleic acid (RNA) viruses, CoV is constantly changing during the mutation process, producing new variants with a high risk of transmission and pathogenicity [13]. New variants can mainly escape the immune response caused by infection and even vaccination [14]. Considering this issue, dealing with this virus and its types in patients with anemia and HDM creates different scenarios and hypotheses or offers suggestions for preventive or therapeutic purposes that should be considered in the treatment and care of affected patients. Because this virus and its variants significantly affect all aspects of human life worldwide, measuring these risk factors among patients with COVID-19 is crucial to help treat the disease. On the other hand, the possibility of false results in diagnostic tests, especially rRT-PCR, has been abundantly observed. According to the research conducted on patients with blood malignancies, there is a higher probability of false negatives, which can depend on endogenous factors such as hematocrit, triglycerides, cholesterol, and other blood substances [15, 16]. Hence, it is necessary to identify the virus in these patients by more sensitive tests such as CRISPR [17]. Therefore, this review article can help young infectious disease specialists, hematologists, and physicians gain a deeper understanding of this condition and quickly look at recent events caused by HDMs.

## COVID-19 in patients with anemia and haematological malignancies

**Fig. 1:**
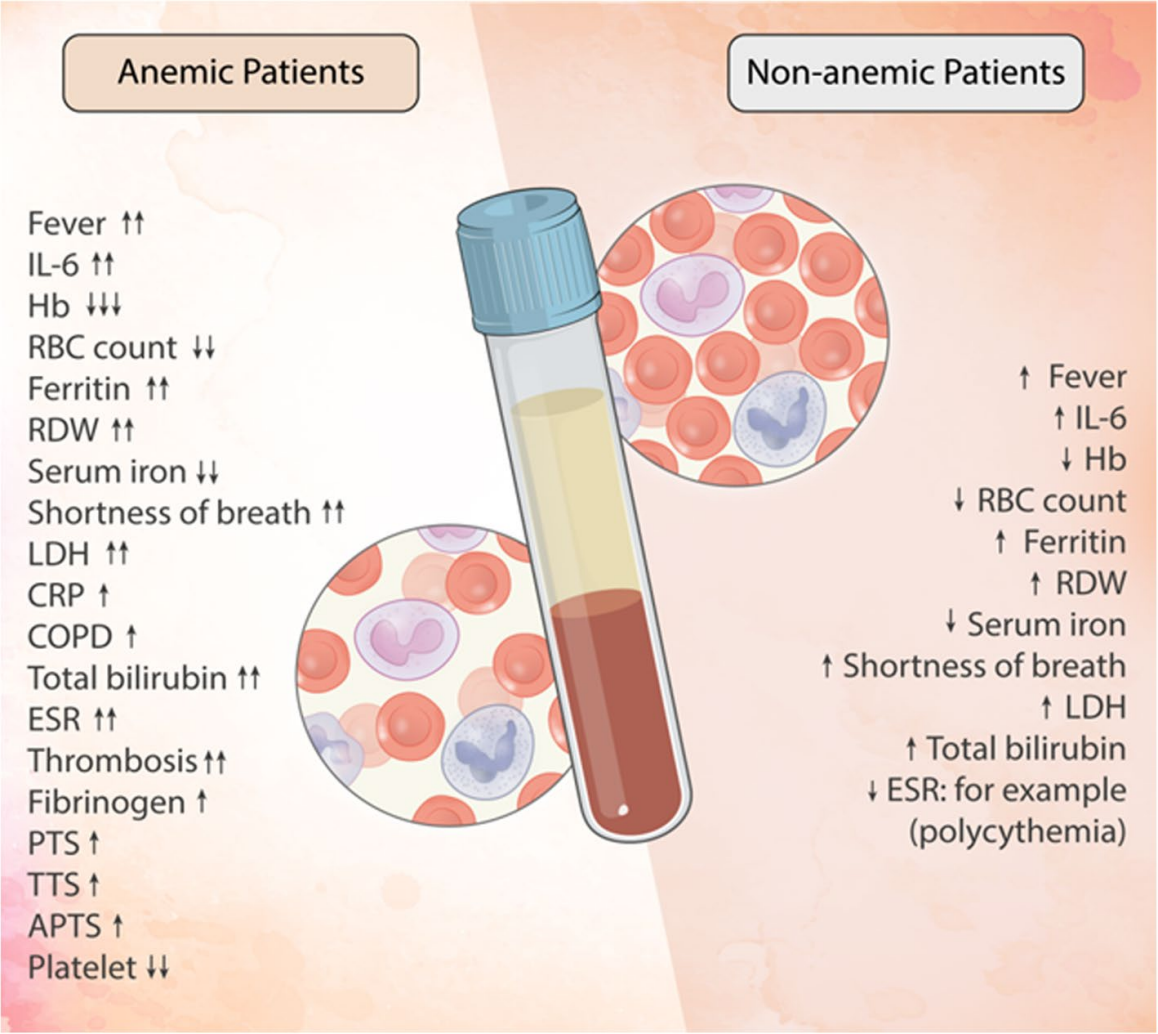
Graphic overview of factors affecting some types of anemia and haematological disorders in the exacerbation of COVID-19

A diverse range of the most common haematological manifestations, including lymphopenia, anemia, thrombocytopenia, hyperferritinemia, coagulopathy, and high D-dimer levels, can be closely related to COVID-19 [18, 19]. The pathogenesis of this condition is mostly attributed to the severe increase of pro-inflammatory markers such as interleukin-1 beta (IL-1β), IL-2, IL-4, IL-6, IL-10, tumor necrosis factor-alpha (TNF-α), and interferon-gamma (IFN-γ) which cause an exaggerated immune response [20]. The inflammatory response also causes disturbances in iron metabolism, contributing to high hepcidin levels, decreased iron utilization, hyperferritinemia, and anemia [21]** (**Fig. 1). Such manifestations can be observed during the infection of COVID-19, which often disappear after the resolution of the infection [22]. Due to the rapid spread of the virus, it is necessary to take necessary precautions regarding the risk factors that make these patients more vulnerable to this disease.

### Beta-thalassemia

**Fig. 2:**
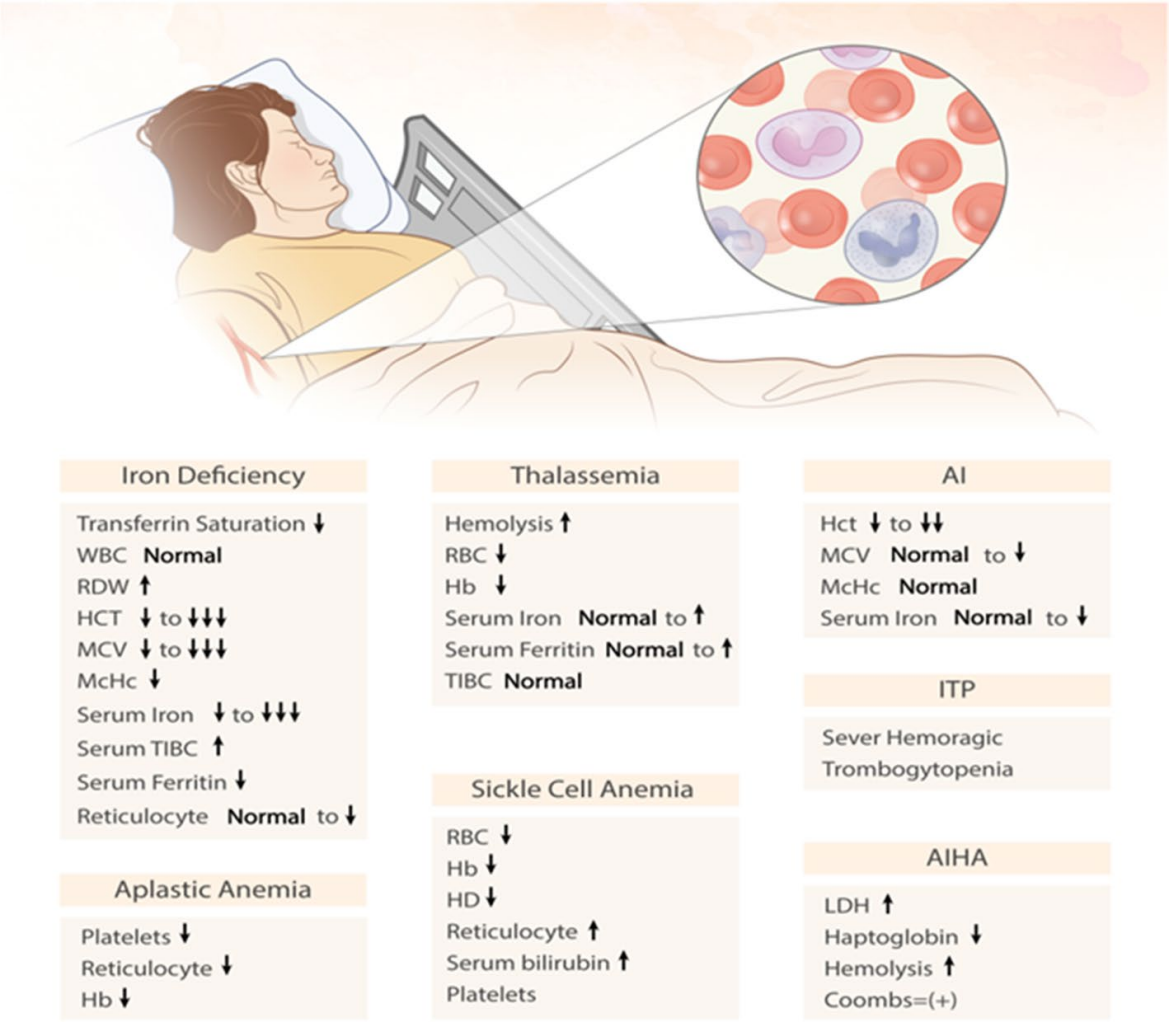
Abnormalities in haematological parameters of patients with anemia and RBC disorders

Beta (β)-thalassemias represent a group of inherited autosomal recessive anemias that occur due to a reduction or absence of beta globulin tetramers (β^4^), also called Hb-H [23]. In cases with β-thalassemia, some polymorphisms of the heme oxygenase-1 (HO-1) gene, especially repeat mutations in the dinucleotide (GT) promoter region, induce the HO-1 gene to generate reactive oxygen species (ROS), which protects the cell [24] ( Fig. 2). In patients with COVID-19, longer GT sequences are likely to be present, resulting in modulation of blood flow [25]. During β-thalassemia (mainly partial form), porphyrin deficiency is not observed, but ferritin and excess serum ferritin [26, 27] and iron have been confirmed as risk factors for the exacerbation of COVID-19 [24].

**Fig. 3:**
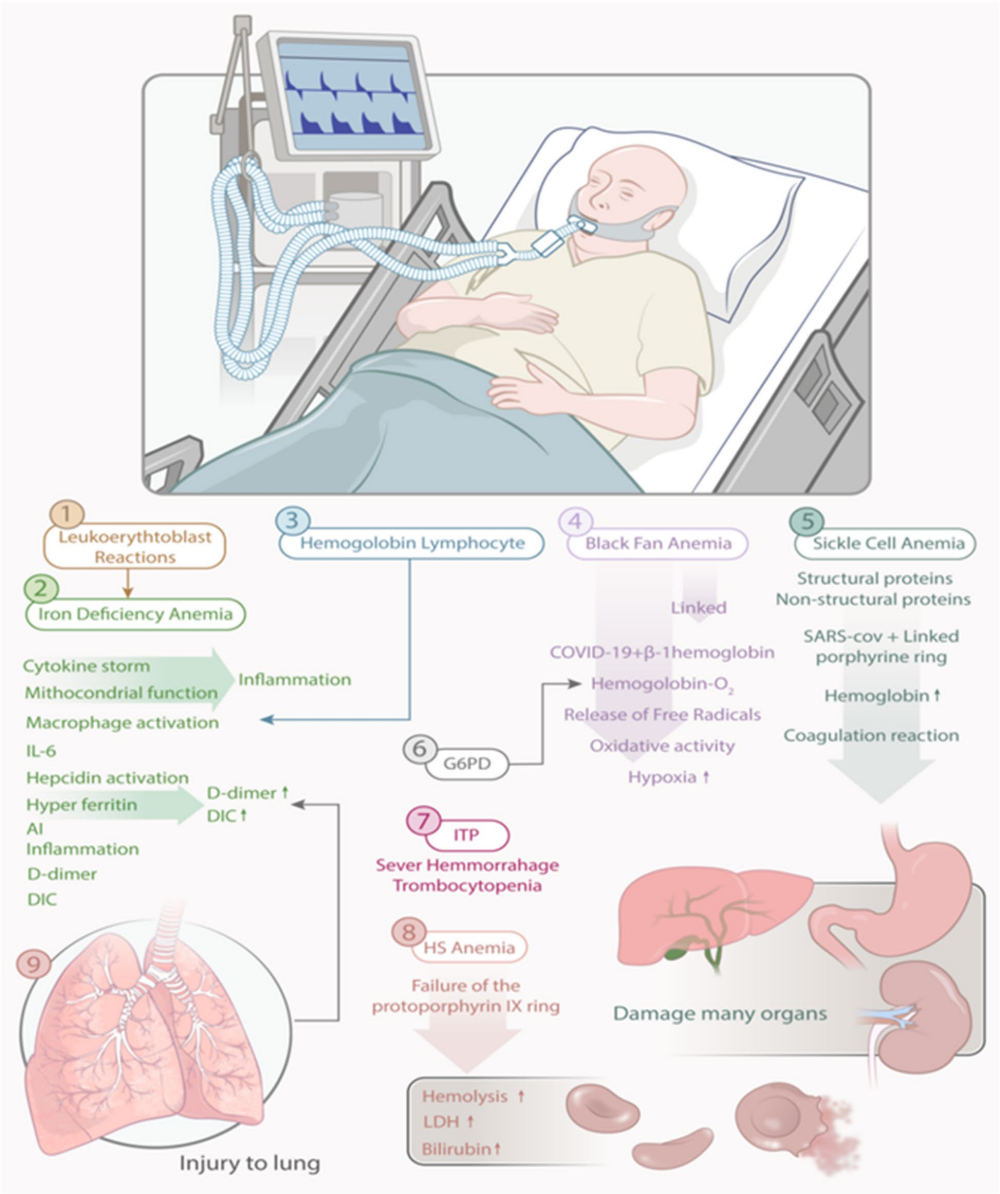
Graphic illustration of the potential role of different types of anemia and blood disorders in the clinical manifestations of people with COVID-19, possibly influencing the pathophysiology of COVID-19

Recent studies have also shown that β-thalassemia patients may contribute to increased susceptibility to SARS-CoV-2 infection due to the nature of their chronic disease [28]. β thalassemias can be defined as an inherited disorder caused by a defect in hemoglobin synthesis, thus accelerating the continuous hemolysis and premature destruction of red blood cells (RBCs) in the bone marrow [29]. These patients are exposed to infectious diseases, especially bacterial infections, acute respiratory infections, and SARS-CoV-2. Therefore, during COVID-19, people with beta-thalassemia are threatened by SARS-CoV-2 infection, as most of them have underlying diseases such as diabetes, common heart diseases, liver problems, and various endocrine disorders [28] (Fig. 3).

### Sickle cell disease

Sickle cell disease (SCD) refers to a type of HDM that has recently been associated with SARS-CoV-2. As confirmed in previous research, people with swine flu (H1N1) commonly develop respiratory complications, including acute chest syndrome (ACS) [30]. Compared to other children with influenza, children with SCD are also referred to health centers much more often [31]. Consequently, such complications are likely to occur more rapidly in patients with comorbid SCD and COVID-19 [32]. Also, SCD leads to an increased chance of several diseases, such as pulmonary hypertension, chronic lung disease, and kidney failure [33]. In this vein, chronic pulmonary injury from thrombo-inflammation caused by COVID-19 exacerbates SCD complications and increases mortality rates [34]. Likewise, acute vaso-occlusive crisis (VOC) attributed to SCD in COVID-19 patients increases the likelihood of pulmonary embolism (PE) and ACS [35]. Thus, individuals with COVID-19 who are co-infected with SCD have a 3.5-fold increased risk of developing PE compared to patients without the disease. Furthermore, the preclinical and procoagulant status of patients with comorbid SCD and COVID-19 may contribute to milder clinical symptoms [36]. According to some clinical studies, patients with SCD and subsequently infected with COVID-19 do not face the risk of complications or mortality from the epidemic. Still, the hospitalization rate is higher in these people, which raises two different hypotheses [37]. First, patients with SCD have severe hemolysis with continuous release of heme, consisting of toll-like receptor 4 (TLR4) with proinflammatory and procoagulant states [38]. In addition, elevated levels of plasma cytokines, such as IL-1, IL-6, and TNF-α, have been reported in individuals with persistent SCD, and S protein up-regulated them for SARS-CoV-2 [39]. As reported in a study, COVID-19 patients with SCD (age > 50 years), presenting a growth in the serum D-dimer, creatinine (Cr), and lactate dehydrogenase (LDH) levels are at greater risk of mortality regardless of their genotype or gender [30]. Accordingly, the pathophysiology of SCD accounts for chronic anemia, endothelial dysfunction, chronic inflammation, immunodeficiency disease, and hypercoagulability, all as risk factors for the worst outcomes of COVID-19 in individuals with a hypercoagulable state and minor pathophysiology during Hypoxia is considered. As with renal modules, such patients could potentially be at increased risk for contracting COVID-19 [38]. Furthermore, people with SCD are likely to develop some neurological complications during their lifetime. Since SARS-CoV-2 adversely affects the central nervous system (CNS) and patients with SCD are completely immunocompromised, many concerns arise that require further investigation [38] **(**Figs. 2 and 3).

### Iron-deficiency anemia

As iron is required for the growth and reproduction of various cells in the immune system, iron deficiency (ID) can impair the host immune response [40]. As an essential trace element for the host, iron is essential for many enzymatic and non-enzymatic reactions as well as various physiological processes [41]. For example, iron significantly contributes to mitochondrial function in adenosine triphosphate (ATP) production or synthesis, RNA and deoxyribonucleic acid (DNA) repair, cell survival, and ferroptosis [42, 43]. In addition, this valuable element is vital for the multiplication of viruses. On the other hand, IL-6 is a key mediator in post-inflammatory iron management, as hepcidin produces iron [44, 45]. As a key regulator of iron homeostasis, hepcidin further destroys the duodenum by damaging the cellular iron exporter, ferroportin (FPN1), which helps promote cell retention in macrophages and regulates cellular iron metabolism. Therefore, inflammation causes some changes in iron homeostasis due to its dysfunction [46, 47]. This deficiency is often compensated by high levels of iron in the reticuloendothelial cells and ultimately by hyperferritinemia, while low levels of iron are present in the bloodstream [48]. Subsequently, inflammation limits iron in RBCs, leading to anemia known as anemia of inflammation (AI), which is commonly seen in pregnant women with decreased red blood cell quantity and quality along with increased erythrocyte sedimentation rate (ESR). It is related to the gas exchange that occurs during the reduction of RBCs [49, 50]** (**Fig. 2). This can be caused by a deficiency of folate and other B vitamins. Therefore, pregnancy when infected with COVID-19, especially in IDA, makes this viral infection more visible in the third trimester because inflammatory processes occur much more often. As reported in some studies, many viruses, including SARS-CoV-2, disrupt iron homeostasis in cells, caused by hemolysis, and then enhance intercellular iron load, which steps it accelerates the multiplication of the virus and ultimately increases the severity of the disease [51, 52]. In any case, this iron overload increases serum ferritin and is further associated with rheumatoid arthritis (RA), multiple sclerosis (MS), antiphospholipid syndrome (APS), macrophage activation syndrome (MAS), adult-onset steel disease (AOSD), catastrophic APS (cAPS), and then septic shock [53, 54] (Fig. 3).

### Aplastic anemia

Aplastic anemia (AA), also known as rare HDM, is characterized by central pancytopenia due to bone marrow failure [55]. Although the pathogenesis of this type of anemia is still unclear, it is hypothesized to result from the destruction of hematopoietic stem cells (HPSCs) secondary to an aberrant immune response [56]. More than 50% of AA cases are also idiopathic in nature [55]. Despite this, chemotherapy (chemo), ionizing radiation, and viral infections also contribute to this disease [57]. Accordingly, the most common infectious agents include viral hepatitis, human immunodeficiency virus (HIV) [58], cytomegalovirus (CMV) [59], parvovirus B19 (PVB19) [60], and Epstein-Barr virus (EBV) [59]. In this regard, SARS-CoV-2 mainly affects the pulmonary system, which in rarer cases leads to central neutropenia, lymphopenia, and pancytopenia and disrupts the hematopoietic system [61]. Moreover, the overproduction of inflammatory cytokines in infectious viruses, such as IL-1ß, IL-6, TNF-α, and IFN-γ [62], disrupts the bone marrow microenvironment and subsequently causes bone marrow failure [63, 64] **(**Fig. 3**)**.

### Diamond-blackfan anemia

Diamond-blackfan anemia (DBA) mainly affects the bone marrow and causes some physical abnormalities in many parts of the body. During DBA, the bone marrow is normally disrupted, resulting in reduced red blood cells to supply oxygen to the tissues [65, 66]. Actually, changes in Hb levels are predictive of worsening clinical progression in patients with COVID-19, as the bone marrow is unable to make RBCs. SARS-CoV-2 can attack the β-1 chain of Hb and detach it from iron to form perforin. Therefore, often less Hb is available to carry oxygen and carbon dioxide (CO_2_). Here, binding of the virus to Hb and subsequent release of ions produces free radicals that increase oxidative stress (OS) in organs and lead to hypoxia. Each Hb molecule also contains four hemes during chemical interactions, each of which binds precisely to oxygen in the lungs [67]. In addition, iron (II) and (III) ions (Fe^2+^ or Fe^3+^), as part of the toxic structure of oxyhemoglobin in a free state, augment OS in the blood. If SARS-CoV-2 binds to Hb Fe^2+^ and Fe^3+^, it may be released into blood and tissues, thus determining the main effects of the virus. In this case, the function of Hb is disturbed, the oxygen supply decreases and finally, hypoxia increases. As a result, shortness of breath and fatigue may persist even after recovery in some patients with COVID-19 [68, 69]** (**Fig. 3).

### Hereditary spherocytosis

In patients with hereditary spherocytosis (HS), cellular stress combined with splenic clearance multiplies the chance of hemolysis [70]. Such individuals may live with chronic baseline hemolysis, sometimes requiring splenectomy to treat severe chronic anemia, or there may be intermittent major hemolysis and splenomegaly [71, 72]. Since the spleen is the site of RBC clearance in HS patients, splenectomy is often advocated as a treatment option [73]. Nonetheless, this surgical procedure does not resolve the defects in the function of the erythrocyte membrane and exposes patients to severe cellular stress and a higher chance of hemolysis [74]. Accordingly, many hemolytic markers can be considered during this emergency. For example, bilirubin (BLR) levels are a significant indicator, the increase of which can be attributed to the breakdown of the protoporphyrin IX (PPIX) ring [70]. Likewise, ferritin is another hemolytic marker, as an acute phase reactant (APR), which is increased in patients with severe COVID-19 with cytokine storm (CS) [75]. Furthermore, LDH levels in severely infected individuals with COVID-19 are compounded due to increased cytokine activity, and decreased monitoring of Hb and hemolytic markers in cases with hemolytic disorders and COVID-19 [76]. Notably, HS can have varying degrees of hemolysis and may be the first hemolytic event at the onset of COVID-19 infection [70].

### Leukoerythroblastic reaction

During the leukoerythroblastic reaction (LER), immature RBCs and myeloid cells often circulate in the peripheral blood [77, 78]. This reaction is commonly reported in some disorders related to bone marrow fibrosis, including myeloproliferative disorders (MPDs) and cancer types associated with bone marrow metastatic problems [79]. LER has been identified mostly in viral infections, such as polycythemia vera (PV) and COVID-19. In severe cases, SARS-CoV-2 infection is associated with overproduction of proinflammatory cytokines such as IL-2, IL-6, IL-7, IL-8, IFN-γ, TNF-α, transforming growth factor-beta (TGF-β), C-X-C motif chemokine ligand 8 (CXCL8), CXCL10, chemokine ligand 3 (CCL3), macrophage inflammatory protein-1 alpha (MIP-1α), and -1β, known as CS. In patients infected with SARS-CoV-2, this condition results in LER, with increased production and the presence of immature myeloid cells in the circulatory system [80]. Leukoerythroblastosis can occur in children with Kawasaki disease. The exact etiology of Kawasaki disease is unknown, although an infectious agent appears to be the source of its initiation [81]. Hypersensitivity reactions or inappropriate immune responses, possibly caused by viruses or bacteria, can trigger an inflammatory process that damages blood vessels in people who are genetically predisposed to the disease. Notably, KD and COVID-19 are very similar in this respect [82–84]** (**Fig. 3).

### Hemophagocytic lymphocytosis

Hemophagocytic lymphocytosis (HLH), introduced as a less common symptom in viral proinflammatory conditions, has a high consequence in most patients with COVID-19 [85]. It is an ambiguous clinical condition, followed by immune-mediated tissue damage, which occurs irregularly due to viral infections or HDM. This phenomenon may not be observed in patients with COVID-19, where phagocytosis is also observed in bone marrow aspirates, cytopenias are present, and serum ferritin elevations below ≥ 2000 ng/mL occur [86]. Also, MAS is a life-threatening proinflammatory syndrome that is likely to appear in patients with severe viral infections, such as those with EBV. There is also an interesting pathophysiological similarity between EBV infection and COVID-19 in the case of MAS. During both infections, uncontrolled and hyperactive macrophages cause hypercytokinemia, organ damage, cytopenias, and coagulopathy [85]. Besides, there are several associations between descriptions of severe forms of COVID-19 infection and secondary HLH (sHLH). For example, elevated serum ferritin and C-reactive protein (CRP) levels are commonly observed in patients with severe COVID-19 and sHLH [87]. Moreover, people with severe COVID-19 develop many complications that resemble multi-organ failure (MOF) in HLH [88]. Since COVID-19 has the same pathogenesis compared to sHLH, early diagnosis, and rapid immunosuppression before MOF are often of particular importance [89]. Therefore, all patients with severe COVID-19 should be screened with standard laboratory tests, such as HScore to detect severe inflammation [90].

### Sideroblastic anemia

Sideroblastic anemia (SA) encompasses a group of inherited and acquired anemias characterized by ineffective erythropoiesis. In this type of anemia, there is an accumulation of ring sideroblasts (RS) in the bone marrow and a decrease in the production of fully developed red blood cells. These ring sideroblasts are nucleated erythroblasts that show abnormal accumulation of iron granules in the mitochondrial matrix [91]. Such mechanisms contribute to the formation of iron-rich mitochondrial complexes around erythroblast nuclei instead of the standard incorporation of iron into PPIX in the mitochondria [92]. It has then again been hypothesized that COVID-19 may induce an immune-mediated genetic defect in a hematopoietic clone, resulting in ineffective erythropoiesis and the development of RS cells [93]. Accordingly, COVID-19 likely induces a genetic change in new genes that cause SA [93]. In addition, SARS-CoV-2 interacts with Hb molecules through a cluster of differentiation 147 (CD147), CD2b, and other receptors commonly found on erythrocytes and other Hb cells, leading to Hb denaturation [94]. Considering that, hemoglobin concentration decreases and toxic heme is released, which usually causes hypoxia [94]. Furthermore, a gradual decrease in the Hb concentration may promote SA and increase erythrocyte distribution width (RDW), indicating overproduction of immature erythrocytes and an increased risk of mortality [95] **(**Fig. 2).

### Megaloblastic anemia

In some cases, megaloblastic anemia (MA), impaired nerve myelin sheath integrity, impaired immune response, neurological complications, and degenerative conditions of the spine can be caused by some effects of low cobalamin levels [96–98]. Following these conditions, symptoms of vitamin B12 deficiency, including elevated OS and LDH, intravascular coagulation and thrombosis, hyperhomocysteinemia, coagulation cascade, subnormal reticulocyte count, vasoconstriction, and renal failure may often accompany COVID-19 [99, 100]. As suggested, high doses of methylcobalamin could potentiate the RNA-dependent RNA polymerase (RdRp) activity of SARS-CoV-2 nonstructural protein 12 (NSP12) enzymes, which then reduces the viral infection and severity of COVID-19. Overall, methylcobalamin helps reduce the severity of COVID-19 [101]. Vitamin B12 deficiency mostly causes two conditions. Sometimes, parietal cells can't make enough vitamin B12 because people don't have a diet rich in this vitamin. In this case, the megaloblast normally forms in the cells and becomes asynchronous when the nucleus and cytoplasm are mixed, also called MA [102]. Accordingly, those suffering from MA do not have enough red blood cells to carry oxygen properly. A second scenario is that vitamin B12 is produced by the parietal cells because a person receives an adequate vitamin B12-rich diet but has difficulty absorbing it [103]. Bacteria in the large intestine also mutate so they cannot absorb vitamin B12 or have challenges in the ion channels that absorb vitamin B12 [104]. To absorb vitamin B12, folate is needed in a trivalent form, and this dihydrofolate is converted from tetrahydrofolate by these colon bacteria with the help of the dihydrofolate reductase (DHFR) enzyme. Therefore, mutations frequently occur that produce this enzyme and lead to pernicious anemia [105]. In an interesting study, vitamin B12 was identified as one of the viral proteins of SARS-CoV-2, so it can easily bind to it and reduce its effects [106]. Therefore, it is necessary to maintain its level. Furthermore, SARS-CoV-2 may interact with the metabolic activities of vitamin B12 and possibly shape the microbiological distribution in the intestine. It occurs if symptoms such as vasoconstriction, increased OS, coagulation cascade, high LDH levels, pulmonary-renal syndrome (PRS), and hyperhomocysteinemia are present. In addition, B12 deficiency can lead to some abnormalities in the CNS, gastrointestinal (GI), and respiratory systems [107]. Accordingly, a recent study has shown that extra doses of methylcobalamin may help minimize organ damage and even some symptoms associated with COVID-19. For example, a study in Singapore showed a significant reduction in existing symptoms of severe COVID-19 in patients taking magnesium, vitamin D (1000 IU), and vitamin B12 (500 μg) supplements [108]. Prenatal pancytopenia is also a rare manifestation, causing anemia, leukopenia, and thrombocytopenia with a simultaneous decline in all blood cell lineages. Vitamin B12 and folate deficiency generally present as MA, but rare manifestations of pancytopenia have been reported so far. The prevalence of vitamin B12 and folate deficiency during pregnancy is currently significantly high in developing countries due to their poor socioeconomic status and nutrition. Hemodilution with interplacental transfer of vitamin B12 further contributes to the physiological reduction of vitamin B12 levels. In addition, pancytopenia is a rare manifestation of some viral infections, including the novel COVID-19 [109].

## Autoimmune and inflammatory haematological complications and COVID-19

As confirmed in related studies, COVID-19 is associated with some autoimmune diseases, including autoimmune cytopenias, cutaneous vasculitis, encephalitis, and Guillain–Barre syndrome (GBS). Among them, autoimmune hemolytic anemia (AIHA) and immune thrombocytopenic purpura (ITP) are the most common [110] **(**Fig. 2).

### Autoimmune hemolytic anemia

In confirmed cases of COVID-19, AIHA or its reactivation has been reported so far, which can be attributed to severe anemia or rituximab treatment. During SARS-CoV-2 infection, anemia associated with elevated LDH and other hemolytic markers may be more frequently observed, and even if the anemia appears unexplained and discontinuous, AIHA is also suspected [111]. Also, molecular mimicry could be the highest factor in the development of AIHA caused by SARS-CoV-2. Immunological cross-reactivity between ankyrin 1 (ANK-1), an RBC membrane protein, and spike proteins in a virus has been implicated in the pathogenesis of AIHA among patients with COVID-19 [112]. Some researchers also believe that the induction of AIHA in children with HLH is due to OS stimulation by SARS-CoV-2. In addition, the acute phase response of COVID-19 induces the formation of aberrant complement immune complexes and complement products on the RBC surface, leading to intravascular thrombosis [113]. This could be consistent with disseminated intravascular coagulation (DIC) with MOF induced by AIHA in COVID-19 patients. Concomitantly, hypercoagulability and inflammatory responses are exacerbated and may affect red blood cells, rupture their membranes, and in such cases lead to PE and vascular coagulation. In this regard, iron and ferritin caused by hemolysis lead to OS. Accordingly, hyperferritinemia and impaired iron homeostasis contribute to endothelial damage and structural changes in red blood cells in cases of COVID-19 [113]. Besides, there are reports of AIHA in patients receiving the vaccination against this disease, particularly with influenza and diphtheria-tetanus-pertussis (DTP) vaccines, due to the induction of warm and cold anti-RBC antibodies [114]. Therefore, vaccines as infectious agents can cause HDMs by molecular mimicry, lymphocyte polyclonal activation, epitope release, and presentation of cryptic antigens [115]. On the other hand, the use of some vaccines cannot protect people with anemia and HDM against SARS-CoV-2 and lead to haematological complications (Table 1).

**Table 1:** Summary of studies showing haematological complications of some post-injection COVID-19 vaccines

Patient	Vaccine or drugs	Results	Ref
A 60-year-old man	Moderna mRNA vaccination	No personal or family history of haematological conditions Bone marrow biopsy confirmed very severe aplastic anemia with severely hypocellular bone marrow	[116]
84-year-old man	Pfizer and BioNTech	One case of severe autoimmune hemolytic anemia was identified in the third week after administration of the Pfizer-BioNTech COVID-19 vaccine. However, the condition improved after corticosteroid treatment	[117]
25-year-old man	Spikevax (mRNA-1273, Moderna Biotech, USA)	Diagnosis of thrombotic thrombocytopenic purpura after SARS-CoV-2 vaccine	[118]
75-year-old woman	BNT162b2	Development of autoimmune hemolytic anemia after vaccination	[119]
69-year-old man	ChAdOx1 and BNT162b2	TTP	[120]
56-year-old male	Pfizer-BioNTech mRNA vaccine	AA	[121]

*Abbreviations*: *AA* Aplastic anemia, TTP, Thrombotic thrombocytopenic purpura

### Idiopathic thrombocytopenic purpura

Depending on viruses and immune and environmental factors, idiopathic thrombocytopenic purpura (ITP) refers to a disease with isolated thrombocytopenia and platelets less than 100 × 109/L, the causes of which are still unknown. Accordingly, autoantibodies reduce platelet synthesis, antibodies against platelet membrane antigens, increase platelet secretion, and prolong life, while platelet production in the bone marrow is reduced due to thrombocytopenia [122]. Acute ITP is usually initiated by a viral infection, and platelet levels usually improve independently after a few weeks or months. Of note, acute ITP lasts more than a year if thrombocytopenia persists** (**Fig. 2). The most potential viruses as triggers are cytomegalovirus, hepatitis C virus (HCV), herpes simplex virus (HSV), varicella-zoster virus (VZV), rubella virus, EBV, influenza virus, HIV, and SARS-CoV. Furthermore, molecular mimicry between virus-specific antibodies and host proteins may cause virus-mediated ITP [123–125]. The main cause of increased mortality in SARS-CoV-2 cases is thrombocytopenia, which can be caused by DIC, thrombosis, septicemia, or drugs. ITP most often occurs during and up to ten days after a COVID-19 infection. In this regard, antibodies directed against viral glycoproteins can interact with platelet surface integrins, such as glycoprotein IIb/IIIa (GP IIb/IIIa) or GPIb-IX-V, which accounts for approximately 5–10% of cases of ITP caused by SARS-CoV-2 [126]. Therefore, patients with COVID-19 and ITP can present with increased thrombocytopenia and excessive bleeding, mainly in the second stage of the disease [127]. According to this, ITP thrombocytopenia is one of the mechanisms that is at the top of the decrease in the number of platelets in patients with COVID-19 [115]. Until now, this phenomenon has been explained for some reasons, especially virus-induced autoimmunity. Therefore, molecular mimicry along with the expression of cryptic antigens or the release of epitopes can clarify this immune disorder. In most cases, ITP can appear two to three weeks after COVID-19 infection and even before vaccination [128] (Fig. 3). On the other hand, it is noteworthy that patients with previous SARS-CoV-2 infection may have excessive procoagulation factors that can lead to thrombosis and thrombocytopenia. However, currently its pathophysiology is unknown. Experimental studies currently show that a type of soluble adenoviral spike protein leads to the formation of thrombosis, which ultimately results from graft events creates significant endothelial inflammatory events, and leads to binding with endothelial cells expressing ACE2 [129].

## Haematological malignancies and COVID-19

**Fig. 4:**
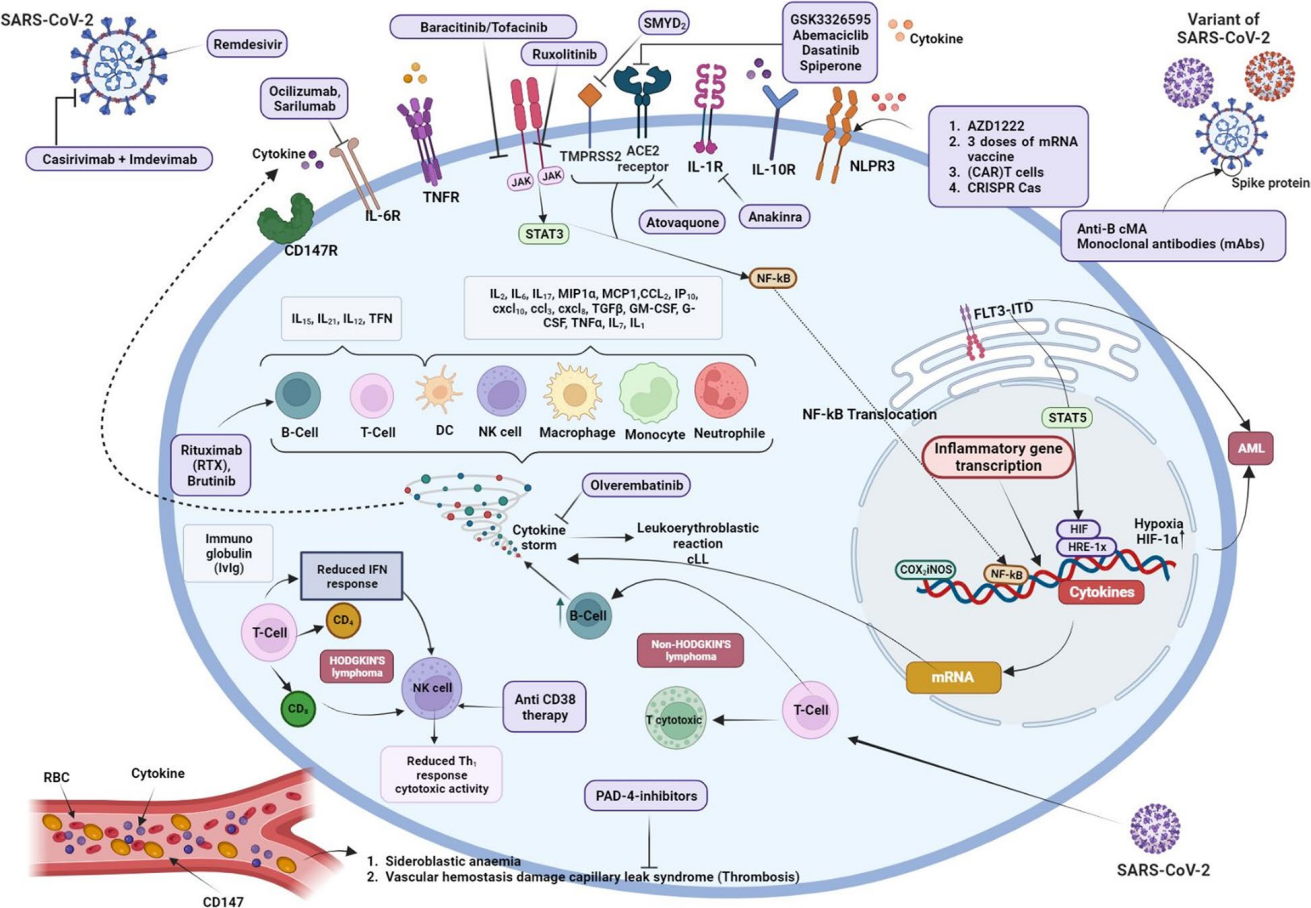
Graphical overview of the effectiveness of different treatments on the mechanisms of patients with haematological malignancies and severe COVID-19. Created with BioRender.com

Patients living with HDM are at increased risk of contracting COVID-19 compared to patients without such symptoms [130]. Such malignancies can affect the production and function of blood cells to fight viral infections [131]. For example, HDM cases often have multiple abnormalities in the innate and adaptive immune system, including low levels of immunoglobulin G (IgG) in patients with chronic lymphocytic leukemia (CLL) or other B-cell neoplasms, as well as immature or neoplastic dysfunctions [132, 133]. Therefore, such immune disorders could make people with HDM susceptible to COVID-19 [134] (Figs. 4 (some are hypotheses and are listed in Tables 4 and 5) and 5 (hypothesis)).

**Fig. 5:**
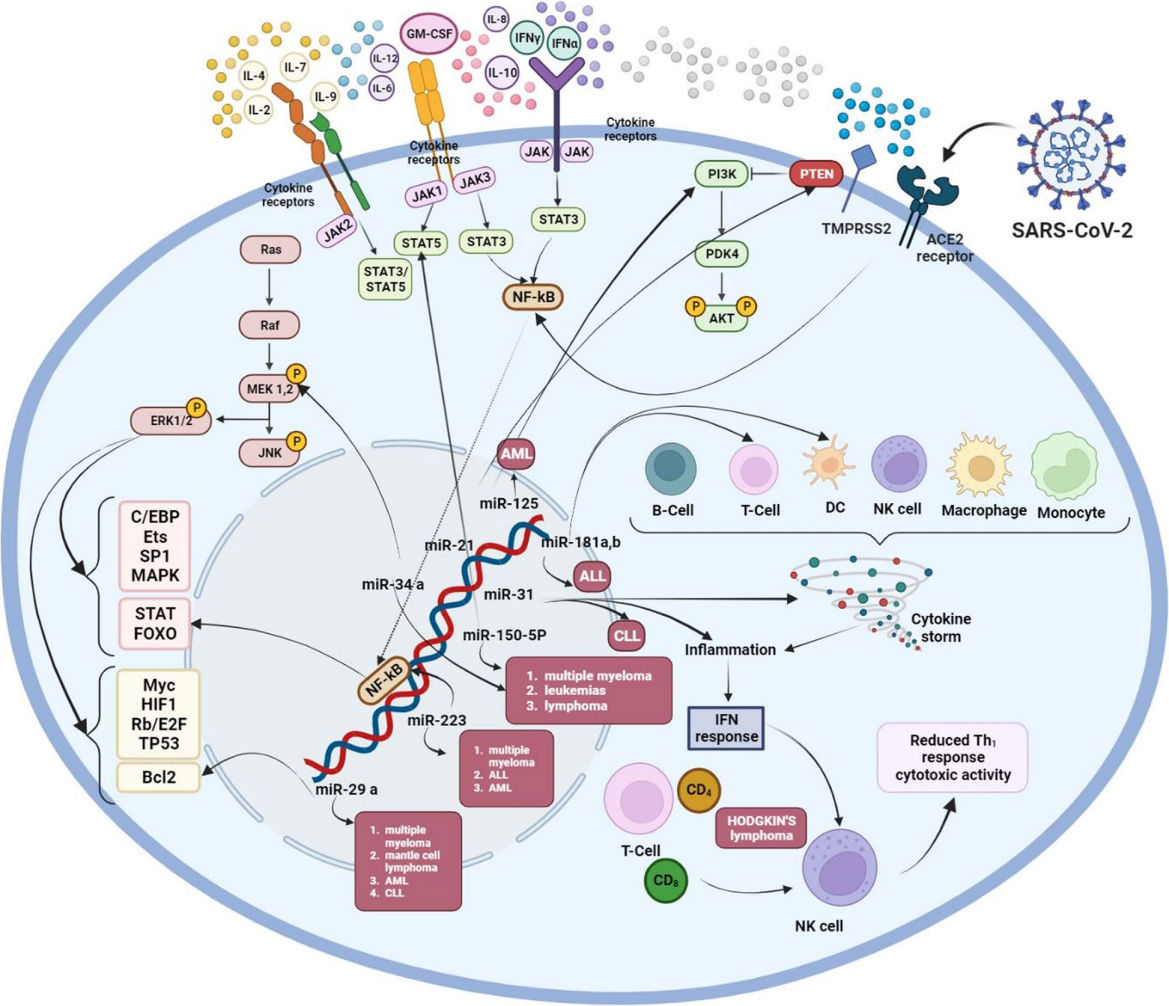
Plausible host miRNA action modes in SARS-CoV-2 infection. Host miRNAs may regulate COVID-19 infection in patients with haematological malignancies. Created with BioRender.com

### Chronic lymphocytic leukemia

As a malignancy, chronic lymphocytic leukemia (CLL) is characterized by an increase in monoclonal CD5^+^ B lymphocytes, leading to intrinsic and extrinsic triggering events [135]. For example, the function of various elements in the immune system during viral infections can determine the onset of CLL [136]. Certain factors such as high levels of markers of immune activation such as IL-4, IL-10, and TNF-α, or cytokine release syndrome (CRS) in patients with COVID-19 and high levels of granulocyte colony-stimulating factor (G-CSF), IL-6, IL-7, IL-8, IL-10, IL-1Rα, IFN-γ, TNF-α, granulocyte–macrophage (GM)-CSF, and monocyte chemoattractant protein-1 (MCP-1) are of great importance in this malignancy. Such cytokines can lead to a rapid increase in the clonal expansion of lymphocytes in COVID-19 patients, potentially increasing the chance of malignancy (Fig. 4) [137]. After cancer patients were infected with SARS-CoV-2, CS could effectively induce a severe form of the disease. In this respect, if the patient is healthy enough, the CS will end and the cancer treatment process will not be interrupted [138]. Therefore, activated signaling pathways may negatively affect the therapeutic response and survival rate in cancer patients just at the beginning and before the end of CS. Accordingly, early detection of CS in such patients (such as patients with CLL) with COVID-19 is critical to multiply the effectiveness of targeted therapy [139, 140]. During acute inflammation, this condition may be caused by high endogenous hormone levels, but additional processes may be beneficial that require further investigation [140]. Moreover, it is not known whether the growth of lymphocyte count is a prognostic marker in patients with severe type of COVID-19 and untreated CLL. Furthermore, similar results are unavailable for CLL subjects who have never been treated. For this reason, treatment for people with COVID-19 and CLL poses great challenges [141]. It also gives the impression that the immune system is ineffective in CLL patients and that lymphocytes do not respond strongly to viral infection. Accordingly, such an agent is likely to help protect these patients against CRS and its subsequent damage and MOF [142]. However, chemotherapy in CLL and COVID-19 cases remains controversial, as it may increase the risk of cardiotoxicity, SARS-CoV-2-induced immunodeficiency, and prognosis [143]. To minimize treatment-induced immunodeficiency and drug interactions, it is therefore best to avoid chemotherapy in patients with comorbid CLL and COVID-19 [144, 145].

### Acute lymphocytic leukemia 

Among the most common types of cancer recognized as the leading cause of death in young adults is acute lymphocytic leukemia (ALL) [146]. Thus, disruption of transcription factors that contribute to direct lymphocyte growth [147, 148], abnormal activation of key signaling pathways, and loss of tumor suppressor genes required for cell cycle regulation [149] are commonly associated with ALL pathogenesis. Furthermore, this condition is often implicated in gene mutations that provide epigenetic regulatory codes [149]. Notably, most cases of ALL and COVID-19 infection have so far not been reported during the pandemic, and the disease progresses slowly in ALL patients with or without clinical symptoms. Therefore, systemic therapy should be delayed in SARS-CoV-2-positive patients (*e.g.*, following the absence of primary hyperleukocytosis). Then, some symptoms such as dry cough, high temperature, anosmia and gastrointestinal problems should be carefully evaluated. If a diagnostic test for SARS-CoV-2 is not possible, a CT scan of the chest should be performed. Furthermore, serological tests should be performed on all patients as soon as they are available [150]. In this line, some studies have further emphasized the abnormally expressed micro (mi)RNAs in ALL patients, as they seem to play a central role in controlling carcinogenesis and drug resistance [151]. Therefore, the etiopathogenesis of HDMs is related to many members of this family, namely miRNA-181a and -181b. Since the expression of miRNA-181a and -181b is much higher in ALL patients than in healthy patients, these findings raise the possibility of using miRNA-181a and miRNA-181b as biomarkers [152]. Researchers have similarly found that all patients living with COVID-19 showed significantly higher levels of miRNA-181a expression, indicating the pathogenic function and prognostic significance of miRNA-181 in patients with comorbid ALL and COVID-19 [153] (Fig. 5 and Table 2).

**Table 2:** COVID-19-associated miRNAs involved in immune response

MicroRNAs	Functions	Types of malignancy	Ref
miRNA-181a and -b	• Regulation of differentiation and development of immune cells and involvement in pathogenesis	ALL	[153, 154]
miRNA-129-2	• It is associated with lung adenocarcinoma and hepatocellular carcinoma through the control of cell growth • In head and neck squamous cell carcinoma with HPV positive and keratinocyte cells transfected with HPV, miRNA-129–2-3p shows increased expression • Given the growing understanding of the relationship between SARS-CoV-2 and comorbidities, changes in miRNA-129–2-3p expression could be of significant importance	Haematological malignancies, including lymphoma	[155]
Hypothesis*			
miRNA-223-3p	• Capable of directly targeting NF-κB inhibitor alpha • Notch and NF-κB signaling pathways increase miRNA-223 transcription and decrease FBXW7 tumor suppressor transcription in T-cell ALL cases	ALL	[156]
miRNA-16 and miRNA-511	• miRNA-16 and miRNA-511 were significantly overexpressed in adult B-ALL	ALL	[157]
MSTRG.119845.30/hsa-miRNA-20a-5p/TNFRSF1B, MSTRG.119845.30/hsa-miRNA-29b-2-5p/FCGR2A, and MST RG.106112.2/hsa-miRNA-6501-5p/STAT3	• Investigating DEGs related to the immune and/or inflammatory response of the host and interaction networks of regulatory genes	Peripheral blood samples of COVID-19 patients	[158]
miRNA-155	• An essential role in the pathogenesis and severity of COVID-19 • Good diagnostic clinical biomarker for diagnosis of COVID-19 disease and severity of infection • A key target in cancer and an ideal target for therapeutic inhibition	DLBCL, AML, ALL and CLL	[159, 160]
miRNA-34a and miRNA-34a-5p	• Up-regulated mRNAs associated with cell proliferation interacted • Decreased expression of more than 30 distinct oncogenes in different cancer pathways (such as MET, MEK1, MYC, PDGFR-α, CDK4/6, BCL2, WNT 1/3, NOTCH1, CD44), along with genes that help tumors evade the immune system response (PD-L1, DGK)	Lymphoma, MM, and leukemias	[161, 162]
miRNA-125	• Related to the formation and growth of tumors, including proliferation of cancer cells, programmed cell death, invasion and dissemination to other parts of the body, metabolic activity, and immune reactions • Acts as an oncogenic factor or tumor suppressor gene and is also associated with drug resistance in various types of leukemia • Manages cervical cancer progression by controlling VEGF and PI3K/AKT signaling pathways	AML	[163, 164]
miRNA-223	• Reduce inflammation to prevent secondary damage during infection and prevent cancerous changes in myeloid cells • Targets of miRNA-223 involved in inflammation and infection include granzyme B, IKKa, Roquin, and STAT3 • In cancer, confirmed targets are C/EBPb, E2F1, FOXO1, and NFI-A • Viral nucleocapsid proteins can suppress BASCs and regulate the release of pro-inflammatory cytokines	AML, MM, and ALL	[156, 165]
miRNA- 150-5p	• It directly inhibits the translation of STAT5b mRNA, which leads to a decrease in total STAT5 protein phosphorylation and can be targeted therapeutically • Overexpression of cellular miRNA-150-5p, which is reduced in COVID-19 patients, can inhibit infection of SARS-CoV-2 target cells • Interaction between cellular miRNA-150-5p and a unique MRE in the NSP10 gene encoded by SARS-CoV-2	MM, AML, malignant lymphoma, Burkitt lymphomas	[166–168]
miRNA-21	• Alterations in miRNA-21 concentrations have the potential to modulate LZTFL1 gene expression, which may ultimately lead to organ fibrosis and inflammatory processes • This may provide valuable insights into the progression of severe symptoms associated with COVID-19 • miRNA-21 is effective in inducing apoptosis in cancer therapies targeting p53-deficient tumors • As miRNA-21 exhibits oncogenic properties, it presents an appealing target for the treatment of MM • Confirmed targets of miRNA-21 (PTEN, Rho-B, and BTG2) are associated with AKT dysfunction and extracellular signal-regulated kinase signaling	Haematological cancers (MM)	[169–171]
miRNA-29a	• Controls ACE2 and may play an important role in the initiation of COVID-19 infection • miRNA-29 participates in a signaling cascade that includes the phosphatase Ppm1d/Wip1, an important modulator of the DNA damage response, and the tumor suppressor p53 • Genes targeted for the regulation of programmed cell death such as the Bcl-2 family of proteins (cellular leukemia/lymphoma) • Dysregulation of the crucial anti-apoptotic protein Mcl-1 is frequently observed in cancerous cells	AML, CLL, MCL, and MM	[172–174]
miRNA-31	• Suppresses the expression of multiple pro-metastatic target genes, thus inhibiting various components of the invasion-metastasis process, including motility, invasion, and anoikis resistance • Significantly up-regulated microRNA in COVID-19 patients was miRNA-31-5p, which could be related to its function in regulating inflammation	CML	[175, 176]

*Abbreviations*: *ALL* Acute lymphoid leukemia, *AML* Acute myeloid leukemia, *BASCs* bronchoalveolar stem cells, *CLL* Chronic lymphocytic leukemia, *CML* Chronic myeloid leukemia, *DEGs* Differentially expressed genes, *HPV* Human papillomavirus, *MCL* Mantle cell lymphoma, *MRE* miRNA recognition element, *MM* Multiple myeloma, *NSP10* nonstructural protein 10, *NF-κB* Nuclear factor kappa B

*Explanations about the function of miRNAs in various haematological malignancies or in COVID-19 raise hypotheses to find a new relationship between COVID-19 and miRNAs involved in the immune response in individuals with haematological malignancies exposed to this virus. It needs more studies and research

### Chronic myeloid leukemia

Chronic myeloid leukemia (CML) is usually initiated by BCR-ABL1 as a hybrid gene in cells with innate or acquired biological potential [177]. This type of cancer can initiate complications in patients with COVID-19 [178]. Furthermore, drug-drug interactions between tyrosine kinase inhibitors (TKIs) for the treatment of this malignancy and those targeting COVID-19 infection may be very hazardous [179]. Also, the side effects of TKI are unbearable for patients with SARS-CoV-2. TKI is often used as an initial treatment for patients with CML and has resulted in a good prognosis and significant improvement [180]. Therefore, treatment with TKIs in cases with CML, which shows a slight increase in the risk of infection, may be due to off-target inhibition of immune-related kinases [181]. Therefore, the decision to withhold or continue TKI-based treatment during the period of COVID-19 seems challenging and needs further investigation. Some studies have also concluded that TKIs help control the immune response to infection [181]. Anti-immune genes such as CD28, CCL55, and IFN-γ and low expression of some such as arginase 1 (ARG1) and fucosyltransferase 4 (FUT4) have been observed so far [182]. Overall, the mortality rate of COVID-19 in Latin American patients with CML has been higher than in the general population. Accordingly, this type of leukemia can cause problems during SARS-CoV-2. Drug interactions between TKIs and COVID-19 treatments can be more dangerous and require careful monitoring [183].

### Acute myeloid leukemia

Acute myeloid leukemia (AML), as one of the most common HDMs, can have adverse effects on blood, bone marrow, and other tissues [184]. This condition is characterized by abnormal proliferation or differentiation of clonal cells and a weakened immune system [185]. Currently, several effective treatments are available for AML, especially for young adults [185]. Therefore, infections, including viral infections, can be a major complication of AML treatment, as in other HDMs. Treatment regimens used for patients with AML can also lead to severe granulocytopenia and an increased risk of serious infections. Therefore, a person with AML and SARS-CoV-2 is at high risk of respiratory failure, which requires a reduction in drug dosage and the fact that antiviral drugs [186].

Besides, it is hypothesized that AML patients with COVID-19 undergo a more severe form of the disease. Some even expire due to these conditions, experience significant progression to recurrence of the malignancy, or become resistant to therapeutic drugs, especially if they harbor FMS-like intronic tyrosine kinase-3 tandem mutations (FLT3-ITD). Based on the theory developed by Zalpoor et al. the pharmacological targeting of autophagy and hypoxia-inducible factor 1 alpha (HIF-1α) may be a potential treatment for FLT3-ITD mutations with COVID-19 and risk of mortality, development of HDMs, and drug resistance [187]. In a remarkable study, Deeb et al. established that cytoplasmic expression of HIF-1α was associated with poor prognosis following conventional therapy in older AML patients with normal karyotype [188]. Therefore, they suggested that stimulation of autophagy and HIF-1α by COVID-19 may be a marker for AML patients, especially those with FLT3-ITD mutations. They also hypothesized that autophagy associated with COVID-19, FLT3-ITD, and overexpression of HIF-1α may cause leukemia and drug resistance in these patients. It probably increases the severity of COVID-19 [188]. However, more studies are still needed to support it. Furthermore, autophagy-related drugs have recently been proposed as potential SARS-CoV-2 treatments based on some in vitro and in vivo studies [189]. Accordingly, it has been hypothesized that autophagy induced by COVID-19 may contribute to cancer growth, chemotherapy resistance, and tumor recurrence in patients. These data also suggest that COVID-19 can induce autophagy due to various factors [190]. In addition to being an antiviral therapeutic strategy, targeting autophagy may be a viable option for treating cancer patients with COVID-19 to reduce the risk of mortality, progression, chemotherapy resistance, and tumor recurrence in a variety of cancers [191] (Fig. 4).

### Multiple myeloma

Another type of HDM is multiple myeloma (MM), which affects plasma cells in the bone marrow [192]. In cases with MM, the immune system is often compromised by various factors making people with this malignancy susceptible to infection [193]. People with a mean age of 65 years have more underlying diseases, so they are at risk of infection [194]. CD4 depletion, lymphopenia, and loss of functional immunoglobulins can increase the chance of viral, bacterial, and fungal infections [195]. Thus, immunosuppressive drugs advocated in this regard can lead to neutropenia, thereby increasing the risk of contracting COVID-19, as the virus exacerbates the cause of abnormally low concentrations of neutrophils in the blood [196]. Notably, new apheresis testing using autologous stem cell transplantation (ASCT) and polymerase chain reaction (PCR) is required before hospitalization in epidemic-affected countries [197]. While living with this anemia, these patients receive treatments that cause some changes in immune system function, such as humoral immunodeficiency, hypogammaglobulinemia, and impaired B-lymphocyte response to SARS-COV-2. Management of MM in the era of COVID-19 accordingly calls for a thorough assessment of patient- and disease-related variables in order to reduce the risk of developing MM through effective treatment [198].

### Myeloproliferative neoplasm

In myeloproliferative neoplasm (MPN), platelets, RBCs, and leukocytes are continuously activated from clonal progenitor cells to hematopoietic cells [199]. PV, ET, and myelofibrosis are also among the leading active neoplasms that can affect mortality [200]. In ET, it is often associated with a persistent increase in the number of platelets that have a propensity for thrombosis, bleeding, and activation of inflammatory mechanisms [201]. In patients with COVID-19 and this malignancy, the lungs may be involved first and then adverse effects on different organs may be observed. In various reports, thrombosis has been presented with some complications of COVID-19 [202]. Therefore, virus-induced thrombosis is a very important genetic thrombosis mechanism in this disease. Accordingly, patients with MPN and COVID-19 are more prone to thrombotic complications and higher mortality [203].

### Hodgkin’s lymphoma

Known as a curable malignancy, Hodgkin’s lymphoma (HL) is probably associated with EBV [204]. First, COVID-19 infection may play a significant role in the transient improvement of HL [205]. Decreased peripheral blood lymphocytes (PBLs) and natural killer (NK) cells can be observed in COVID-19 patients [206, 207]. As well, the total number of lymphocytes (here, the CD4^+^ and CD8^+^ cells) decreases in severe forms of the disease, more than in mild cases [208]. Second, inflammatory microenvironments may minimize the effective function of NK cells. The high levels of IL-6 and IL-10 in these patients therefore add to the capacity to reduce the cytotoxic process and increase the expression of NKG2A in killing virus-infected cells [205]. In these patients, it also binds to angiotensin-converting enzyme 2 (ACE2) in NK cells and suppresses their function [205]. SARS-CoV-2 and subsequent immune cell inflammatory responses inhibit NK cell cytotoxicity, induce CRS, and amplify inadequate immune responses [209]. Third, individuals with EBV-positive HL is that the NSP10/NSP7/3CL^pro^/major protease (M^pro^) and SARS-CoV-2 S proteins bind to the tumor necrosis factor receptor type 1-associated death domain protein (TRADD) at the binding site of latent membrane protein 1 (LMP1), blocking LMP1 binds to TRADD. This interaction may therefore inhibit LMP1-mediated nuclear factor kappa B (NF-κB) signaling to induce remission [206] (Figs. 4 and 5).

### Non- Hodgkin's lymphoma

The most common type of cancer in HIV-infected individuals is non-Hodgkin's lymphoma (NHL), with an increased incidence of B-cell aggressive NHL [210]. Factors affecting the emergence and development of NHL include HIV infection with high viremia, the presence of EBV, and possibly SARS-CoV-2 infection [210]. Recent data also suggest that patients with HDM, including patients with B-cell NHL, are at high risk of severe COVID-19 and may act as a persistent viral reservoir that gives rise to new and potentially more aggressive mutations. Therefore, prevention of COVID-19 or at least modulating its severity in these patients is of great importance [211]. Moreover, patients with B-cell NHL have lower rates of seroconversion and antibody levels compared to other subjects with HDM. Patients with B-cell NHL are also at increased risk of complications and mortality from SARS-CoV-2 [212]. Vaccination against SARS-CoV-2 reduces COVID-19-related deaths and hospitalizations. However, NHL cases experience suboptimal antibody responses to COVID-19 vaccines before and after B-cell-targeted therapies, such as the rituximab anti-CD20 antibody therapy [213] (Fig. 4).

### Overview of therapeutic candidates for COVID- 19 infection and related variants

The current pandemic of SARS-CoV-2 and COVID-19 has so far resulted in high rates of mortality and morbidity worldwide. Hematology societies are therefore suggested to conduct prospective and multicenter studies to clarify the effects of this virus and even measure disease severity in patients with anemia and HDMs [214]. In this regard, various blood markers that act as prognostic markers in the severity of the disease have been investigated in previous researches. In this case, patients with HDM are at a higher risk of contracting various infections, including SARS-CoV-2 [215] (Table 3).

**Table 3:** Summary of studies evaluating the impacts of COVID-19 in patients with anemia and haematological malignancies

Country	Time of study	Patient	Results	Type of malignancy	Ref
Italy	Feb 25 and May 18, 2020	• 198 (37%) of 536 patients died • Compared to the general Italian population with covid-19, the standardized mortality ratio was 2.04 in our entire study group and 72.3 in people younger than 70 years • Compared to the non-COVID-19 group with haematological malignancies, the standardized mortality ratio was 41·3	People with haematological malignancies experience worse outcomes compared to the general population with COVID-19 and patients with haematological malignancies who are not infected with COVID-19	Leukaemias, myelodysplastic syndromes, myeloproliferative neoplasms, lymphomas, and MM	[214]
Turkey	11 March 2020 and 22 June 2020	• COVID-19 patients with haematological malignancy (*n* = 740)	Patients with haematological malignancies are at increased risk of experiencing severe COVID-19 events, such as ICU admission, MV support, or death, compared with non-malignant COVID-19 patients	NHL (30.1%), myelodysplastic syndrome (19.7%), and myeloproliferative neoplasm (15.7%) were the most common haematological malignancies	[215]
China	Jan 13 and Mar 18, 2020	• 205 patients with cancer and laboratory-confirmed SARS-CoV-2 infection and 22 patients (11%) had haematological malignancies	Patients with cancer and COVID-19 who were hospitalized had a high mortality rate	Haematological malignancies	[216]
Spain	March 7, 2020, and April 7, 2020	• Mortality of patients with haematological malignancies compared to non-cancer patients (35.9% vs. 13.2%)	Mortality from COVID-19 is significantly higher in patients with haematological malignancies compared to non-cancer patients	Lymphoma (30%) and MM (30%)	[217]
Italy	March 1, 2020, and April 11, 2020	• 206 patients with COVID-19 and the prevalence of anemia in COVID-19 was 61%	COVID-19 often leads to anemia as a widespread symptom. While anemia may not have a direct impact on mortality, it can significantly affect the well-being of the elderly and vulnerable, thereby reducing their quality of life	Non-malignant	[218]
China	1 December 2019 to 20 March 2020	• 222 confirmed patients including 79 patients with anaemia and 143 patients without anaemia	Anemic COVID-19 patients showed higher rates of comorbidities, more severe inflammatory responses, and organ damage compared to non-anemic controls	Non-malignant	[219]
Italy	May 01 and June 15, 2020	• 860 patients with malignancy were tested, of which 474/860 (55%) had haematological malignancies and 386/860 (45%) had solid tumors	Increased risk of contracting COVID‐19 in patients with haematological malignancy	Neoplasms were lymphomas in 198/860 (23%) cases, breast cancer in 103/860 (12%) cases, MM in 103/860 (12%) cases, acute leukemia in 83/860 (9.5%) cases, and lung cancer in 81/860 (9%) cases	[220]
Japan	Feb-22	• The average (± SD) age of 9 individuals was 74 ± 7 years (ranging from 61 to 85 years), and 6 people (66.7%) were male. All participants had a case of ongoing HM: 3 (33.3%) with myeloproliferative disorder. 6 (66.7%) of the patients had received two vaccines more than half a year ago	Although the Omicron strain can be more serious than previous cases, the mortality rate for hospitalized people with underlying health conditions who are infected with the Omicron strain is still significant	4 (44.4%) with malignant lymphoma, and 2 (22.2%) with MM	[8]

*Abbreviations*: *MM* Multiple myeloma, *NHL* Non-hodgkin’s lymphoma

On the other hand, this study is very important in the management of patients with blood cancers in the face of SARS-COV-2 and its variants. It also emphasizes the priority of these patients in receiving vaccines and many other treatments. Also, various vaccines and treatment methods SARS-COV-2 and its variants have been considered in different patients (Tables 4 and 5).

**Table 4:** Potential therapeutic candidates for immunomodulation in COVID-19 infection

Therapeutic candidates	Types of vaccines or drugs	Haematological malignancies	Description (advantage, disadvantage, function, or target)	Ref
BNT162b2	mRNA vaccine	Lymphoma patients	BNT162b2 vaccine induces a significant humoral response in a significant proportion of patients with B-cell non-Hodgkin's lymphoma (B-NHL) regardless of gender or age. However, patients receiving active treatment with anti-CD20 antibodies experience impaired humoral responses. In fact, it is impossible to achieve a humoral response in the first 9 months after anti-CD20 therapy. However, response rates gradually improved after this period and continued to increase over time.	[221]
mRNA-1273 (Moderna)	Nanoparticle–encapsulated mRNA vaccine	Lymphoma patients	Vaccination with mRNA-1273 or Ad26.CoV2.S prime vaccination, as well as the mRNA-1273 booster compared to BNT162b2, resulted in higher levels of antiviral antibodies in patients with HM, (haematological malignancies), unlike the similar efficacy observed in healthy individuals. These differences in immunogenicity could be attributed to variations in spike mRNA quantity, coding sequence, lipid composition of vaccines, and dosing schedules.	[222, 223]
Recombinant subunit zoster vaccine^*^	Recombinant subunit	B-NHL	• Prevention of HZ in adults over 50 years of age • Provide immunity in a significant proportion of immunocompromised adult patients over 18 years of age, while maintaining an acceptable safety profile	[224–226]
Johnson & Johnson^*^	Adenovirus based vaccine	N.A	• A single vaccination was up to 66% successful in preventing moderate to severe COVID-19, while completely preventing hospitalization and death from COVID-19 • There were no reports of severe hypersensitivity or adverse reactions to vaccination	[227, 228]
Dexamethasone^*^	Small-molecule inhibitor	CLL	• A glucocorticoid drug	[229]
Baracitinib/tofacinib^*^	Small-molecule inhibitor	Pyoderma gangrenosum	• JAK–inhibitor	[230]
TKI^*^	Small-molecule inhibitor	CLL	• Tyrosine kinase	[231]
Hydroxyurea therapy^*^	Small-molecule inhibitor	SCA and CML	• Increases fetal hemoglobin and reduces the number of attacks • Inhibiting the enzyme ribonucleotide reductase by scavenging tyrosyl free radicals decreases the production of deoxyribonucleotides as these radicals play a role in reducing NDPs	[232]
Remdesivir^*^	Small-molecule inhibitor	CLL, Lymphoma	• It is a nucleoside-like compound that inhibits the RdRp of coronaviruses • Dynamics of temperature, C-reactive protein, and lymphocyte count indicate SARS-CoV-2 reinfection	[233]
Ibrutinib^*^	Small-molecule inhibitor	N.A	• XLA • MCL • CLL	[234]
Ruxolitinib^*^ (Jakafi, Incyte)	Small-molecule inhibitor	N.A	• Myelofibrosis	[235]
Venetoclax^*^	Small-molecule inhibitor	SLL, CLL and AML	• Bcl-2 inhibitor	[236]
RTX^*^	Anti-CD20 Monoclonal antibody	Non-Hodgkin's B-cell lymphoma and rheumatoid arthritis	• This results in the removal of CD20 from the cells, allowing them to persist and resist clearance • Used to treat haematological and autoimmune diseases by depleting CD20-expressing B-cells	[237]
Casirivimab/Imdevimab^*^	Monoclonal antibody	Leukemias, myelodysplastic syndromes, myeloproliferative neoplasms, lymphomas, and MM	• A mixture of two MABs neutralizing human IgG1 against SARS-CoV-2 spike protein	[238, 239]
Tocilizumab, sarilumab^*^	Human monoclonal antibody	HLH	• Anti-IL-6	[240]
Temelimab^*^	IgG4 monoclonal antibody	N.A	• Increased intensity of HERV-W envelope protein expression in leukocytes of COVID-19 patients • HERV-W is a potential biomarker in severe cases of COVID-19 • Temelimab targets the HERV-W^**^ protein and can be investigated as an option to reduce the severity of COVID-19 in patients with blood malignancies	[241, 242]
Isatuximab/daratumumab^*^	Anti-CD38 antibody	MM, CLL, AML, etc	• Allosteric kinetic inhibitors of CD38	[243]
hATG^*^	An infusion of horse or rabbit-derived antibodies against human T cells and their precursors	Severe acquired aplastic anemia and AML	• The use of hATG in AML patients receiving intensive ventilation had no significant adverse effect on clinical outcomes	[244]
HCT and cell therapy^*^	N.A	Many inherited or acquired disorders of the hematopoietic system such as ALL	• High safety and low toxicity data from patients treated with allogeneic HCT after receiving CAR-T therapy	[245]
Convalescent plasma therapy^*^	N.A	ALL	• Shorter hospital stay and lower mortality	[246]
IG^*^	Mediated by the Fe portion of IgG and by the spectrum of variable (V) regions contained in the immune globulin preparations	Malignant lymphoma and MM idiopathic thrombocytopenic purpura, Kawasaki disease, Guillain-Barré syndrome, dermatomyositis	• Immunomodulatory properties • Involved in association with T cell surface molecules critical for immune regulation, such as αβ TCR, CD5, CD4, invariant components of MHC class I molecules, and T- and B-cell adhesion molecules • Increased platelet count	[247]
AZD1222^*^	Replication-deficient simian adenovirus expressing SARS-CoV-2 spike protein	WM, CLL, and NHL	• Neutralization of antibodies and antigen-specific T cells against SARS-CoV-2 spike protein • After the first vaccine dose, WM/CLL/NHL patients had lower neutralizing Ab titers than controls	[248, 249]
Anakinra^*^	Recombinant human IL-1 receptor antagonis	HLH	• Anti-IL1	[250]

*Abbreviations*: *ALL* Acute lymphoid leukemia, *AML* Acute myeloid leukemia, *B-NHL* B-cell non-Hodgkin lymphoma, *CAR* Chimeric antigen receptor, *CLL* Chronic lymphocytic leukemia, *CML* Chronic myeloid leukemia, *DVM* Donor versus malignancy, *DVR* Donor versus recipient, *HCT* Hematopoietic cell transplantation, *HSCT* Hematopoietic stem cell transplantation, *HLH* Hemophagocytic lymphohistiocytosis, *HZ* herpes zoster, *Hatg* Horse anti-thymocyte globulin, *IG* Immune globulin, *IgG* Immunoglobulin gamma, *IL* Interleukin, *MCL* Mantle cell lymphoma, *NHL* Non-Hodgkin lymphoma, *NDPs* Nucleoside diphosphates, *RVD* Recipient versus donor, *RTX* Rituximab, *RdRp* RNA-dependent RNA polymerase, *SCA* Sickle cell anemia, *SLL* Small lymphocytic lymphoma, *WM* Waldenstrom Macroglobulinemia, *XLA* X-linked agammaglobulinemia

^*^Hypotheses about the use of vaccines and other treatments, as well as the possibility of finding new treatment methods in people with blood diseases exposed to this virus, have been proposed with further studies

^**^It is important to mention that with the progress in treatments, HERVs can be part of side effects related to treatment and drug resistance mechanisms. For example, aberrant transcription of MDR-1 by ERV1 LTR MER57 found in lymphoma cells is a clear example. As a result, to reduce the transcription of harmful genes, several inhibitors of DNA methyltransferase (DNMT) and histone deacetylase (HDAC) are used in the treatment of malignancies

**Table 5:** SARS-CoV-2 variants in patients with haematological malignancies: summary of received treatments and hypothesis for haematological malignancies

Types of vaccines or drugs	SARS-CoV-2 variants	Haematological malignancies	Description (advantage, disadvantage, function, or target)	Ref
**Drugs**				
AZD7442 (tixagevimab–cilgavimab)	Omicron	NHL B-cell malignancies	• AZD7442 did not successfully neutralize Omicron-RBD in patients with haematological malignancies who received a single dose of 150 mg • Neutralization increased above the positive threshold after a 300 mg dose, although it was still variable • AZD7442 counteracts weak performance against contemporary Omicron SARS-CoV-2 strains	[251–254]
Dexamethasone	Omicron	Myeloma	• Administration of dexamethasone resulted in a reduction in 28-day mortality in subjects randomized to receive oxygen therapy or invasive mechanical ventilation. No significant reduction in mortality was observed in patients who received no respiratory support	[8, 255, 256]
Plitidepsin	SARS-CoV-2 B.1.1.7	Haematological malignancies	• It inhibits the translation of ORFs, ORF1A and ORF1B, leading to reduced synthesis of pp, pp1a and pp1ab, and through eEF1A drive, reduces the amount of repetitive nonstructural proteins such as RNA-dependent RNA polymerase • Inhibits the translation of various sub-genomic mRNAs, resulting in insufficient production of structural and auxiliary viral proteins • The absence of essential viral proteins such as RdRp and structural proteins simultaneously prevents the production of virus copies	[257–259]
Remdesivir and plasma therapy and free remdesivir	SARS-CoV-2 variants and Omicron	Acute B Lymphoblastic leukemia, AML, ALL, NHL, myeloma/plasmacytoma, myelodysplastic syndrome, CLL, active neoplasia	• Accelerate resolution of infection and safe initiation of immunosuppressive therapy • It is a nucleoside-like compound that inhibits the RdRp of coronaviruses	[260, 261]
Bamlanivimab D etesevimab	Alpha variant	B-cell malignancies	• Causes SARS-CoV-2 immune escape mutations and secondary clinical deterioration in COVID-19 patients with B-cell malignancies	[262]
Obatoclax^*^	Alpha (B.1.1.7), Beta (B.1.351), and Delta (B.1.617)	N.A	• Block endocytosis and membrane fusion	[263]
Olverembatinib	Omicron	CML	• Inhibits the release of cytokines	[264]
Abemaciclib, Dasatinib and Spiperone^*^	Omicron	N.A	• Block the interaction between the omicron spike protein and the host cell receptor ACE2	[265]
Azacytidine^*^	Delta	N.A	• As a potent inhibitor of DNA methylation, both in preclinical models and in cancer patients	[266]
Atovaquone^*^	SARS-CoV-2 and other variants of concern including the alpha, beta, and delta variants	N.A	• Inhibits replication • Its ability is partly related to the expression of TMPRSS2, and the drug can prevent the spike protein from binding to the viral receptor, ACE2 • Spike-mediated membrane fusion was also reduced in the presence of atovaquone	[267]
Venetoclax (BTK inhibitors)	Delta	AML, CLL, SLL, B-cell malignancies, and MCL	• Reduction of mortality • Antitumor agents	[268, 269]
**Vaccines**				
BNT162b2^*^	Alpha and Delta	MM	BNT162b2 induced a specific T cell response in healthy individuals. Conversely, in MM patents, T cell responses are weaker and more heterogeneous than healthy controls.	[270, 271]
3 doses of mRNA vaccine	SARS-CoV-2 variants expect Omicron	CLL	• Robust hybrid immunity in serum, saliva, and T-cell compartments in patients	[272]
mRNA-1273	Omicron	MM	• MM patients who received mRNA-1273 vaccination developed severe infections 10 weeks after vaccination, despite the production of protective antibodies • After administration of two mRNA vaccines to people with NSCLC and healthy participants, it was observed that NSCLC patients showed less neutralizing activity against live viruses compared to their healthy counterparts	[273]
Moderna (second vaccine) Pfizer (second vaccine) Janssen (1 vaccine)	Omicron	AML	• Boosted immunity	[274]
**Monoclonal antibodies**				
Anti-CD38 therapy	Alpha and delta	MM	• It is approved in the first line in combination with other agents (immunomodulatory drugs—IMID or PI) and high-dose steroids • Anti-CD38 therapy was even less associated with NAbs response among MM patients • Anti-CD38 antibodies also cause a relative depletion of NK cells, which could contribute to immunodeficiency in MM receiving these immunosuppressive regimens	[275]
Anti-CD20	SARS-CoV-2 (delta)	B-cell malignancies	• Disrupts humoral responses after two or three vaccinations • Patients who received anti-CD20 antibodies showed limited efficacy of a BNT162b2 booster dose	[276–279]
Anti-BCMA MAbs	Omicron, WA1, and delta	MM	• After the third time, no significant increase in anti-spike Ab levels was observed in patients treated with anti-BCMA MAbs	[280]
ACE2-blocking antibody	Omicron BA.1 and BA.2 and other SARS-CoV-2 variants	Haematological malignancies	• Maintain potent neutralization and protection against Omicron and other SARS-CoV-2 variants	[281, 282]
Antibody-containing plasma	Omicron variant	Haematological malignancies	• Control virus replication	[283]
CAR-T cells	SARS-CoV-2 variants (Omicron variant)	CLL B-cell-depleted lymphoma	• Vaccine-induced T-cell response against SARS-CoV-2 and its Omicron variant in patients • CD19-CAR-T therapy selectively depleted all CD191/CD201 B-cells from the blood of our patients, thus eradicating the immune cell compartment secreting anti-SARS-CoV-2 antibodies	[280, 284]
Cellular therapies	SARS-CoV-2 variants	Haematological malignancies	• Cell therapy used by adoption to prevent or treat viral infections in cases of natural or transplant immune errors is safe and effective against herpes viruses, polyomaviruses, and certain respiratory pathogens such as adenovirus • Adoptive T-cell therapy has been investigated as a prophylactic or curative adjuvant therapy against SARS-CoV-2	[285–287]
GSK3326595^*^	Omicron, delta, and beta variants	N.A	• By inhibiting ACE2-R671 dimethylation • Able to significantly reduce ACE2 binding to RBD • Strongly reduces the interaction of ACE2 with Spike1	[288]
T-cell or B-cell immunotherapy	Omicron	Haematological malignancy (MM, lymphoma, CLL, and etc*.*)	• Low levels of nAb after two or three doses of vaccination • SARS-CoV-2 variants showed partial vaccine escape, especially the Omicron variant (BA.1) compared to the Delta variant (B.1.617.2) • Resistance to anti-SARS-CoV-2 mAb	[289]
ALVR109^*^	Alpha, Beta, Gamma, Delta, Epsilon, and Kappa	N.A	• On day 10 after the second injection of ALVR109, the patient`s nasopharyngeal SARS-CoV-2 viral load became persistently untraceable • After the second infusion, the patient was discharged on minimal oxygen support and received a second negative result of quantitative nasopharyngeal PCR	[290]
CAR-NK cell therapy^*^	SARS-CoV-2 variant	Leukemia and MM	• CAR-NK cells target SARS-CoV-2 spike protein with CR3022 scFv domain, a potent neutralizing antibody targeting SARS-CoV-2 S protein • The results showed that CR3022-CAR-NK cells can kill SARS-CoV-2 infected cells in vitro	[291]
**Inhibitors**				
PAD-4 inhibitors	SARS-CoV-2 variants	MM	• Offers broad therapeutic potential in a wide range of inflammatory diseases such as COVID-19 through the formation of NETs • In reducing thrombotic complications in various inflammatory disorders such as COVID-19 and suggests that these inhibitors may be valuable in the treatment of immunothrombotic origin of SARS-CoV-2	[292]
FLT3 inhibitors	SARS-CoV-2 variant	AML	• Decreased autophagy can reduce the risk of chemotherapy resistance and mortality	[293]
SMYD2^*^	Delta variant	N.A	• Inhibition downregulates TMPRSS2 • Reduces SARS-CoV-2 infection	[294]
**Other treatment**				
Convalescent plasma	Alpha	B-cell lymphoid malignancy AML	• Reduction of mortality	[295, 296]
Nanobodies^*^	Emerging mutant variants like Alpha (B.1.1.7), Beta (B.1.351), and Gamma (P.1)	N.A	• Exclusive neutralization efficiency	[297]
CRISPR-Cas^*^	SARS-CoV-2 variant	T cell ALL	• Development of global T cells against COVID-19 • Enormous potential for the advancement of CAR-NK cell therapy through the use of CAR-NK cells derived from induced pluripotent stem cells. This is due to their ability to manipulate genes and cell surface receptors that have already been studied in the development of CAR-T cells or genetic mutations that cause disease	[291]

*Abbreviations***:** *ALL* Acute lymphoid leukemia, *AML* Acute myeloid leukemia, *BTK* Bruton’s tyrosine kinase, *CAR* Chimeric antigen receptor, *CLL* Chronic lymphocytic leukemia, *CRISPR* Clustered regularly interspaced short palindromic repeats, *CML* Chronic myeloid leukemia, *MCL* Mantle cell lymphoma, *MM* Multiple myeloma, *NK* Natural killer, *NETs* Neutrophil extracellular traps, *nAb* Neutralizing antibodies, *NHL* Non-hodgkin’s lymphoma, *NSCLC* non-small cell lung cancer, *ORFs* Open reading frames, *PCR* Polymerase chain reaction, *pp* Polyproteins, *PI* Proteasome inhibitors, *RBD* Receptor‐binding domain, *RdRp* RNA-dependent RNA polymerase, *SLL* Small lymphocytic lymphoma

^*^Hypotheses about the use of vaccines and other treatments, as well as the possibility of finding new treatment methods in people with blood diseases exposed to this virus, have been proposed with further studies

## Conclusion and future directions

In conclusion, COVID-19 has presented unique and significant challenges for patients with anemia and hematological malignancies, who face a higher risk of severe illness and mortality. This review article has examined the risk factors, clinical guidelines, and emerging therapeutic approaches for managing COVID-19 in this patient population. While much progress has been made in understanding COVID-19 in this context, there are still many areas that require further research.

Prospective type comparative studies using different vaccines, drugs, or combinations against SARS-CoV-2 and its multiple variants in HDM cases are necessary to discover the best options for this specific scenario. Considering the effects of anemia and HDMs on the quality of human life, this issue cannot be ignored, especially during the COVID-19 pandemic, and even because of the high costs, side effects, and shortage of blood. It is hoped that knowing the types of anemia and HDM in the face of this virus, as well as the vaccines and drugs used for the virus itself and related types, will help reduce the clinical burden of COVID-19 and its variants in terms of treatment and care.

For future directions, researchers must focus on uncovering the long-term effects of COVID-19 on patients with anemia and hematological malignancies. Specifically, understanding the cellular and molecular mechanisms of SARS-CoV-2 in potential long-term effects such as chemotherapy resistance, metastasis, and recurrence can open new avenues for developing therapeutic and preventive strategies. In addition, future studies should evaluate the efficacy and potential side effects of vaccination and emerging therapeutic approaches, with a focus on developing new vaccines and drugs that are more suited to the clinical, cellular, and molecular conditions of these diseases to improve efficacy and reduce side effects.

This study provides a potent foundation for preparing for future outbreaks of newly emerged coronaviruses. By examining the risk factors, clinical guidelines, and emerging therapeutic approaches for managing COVID-19 in this patient population, we can gain valuable insights into the challenges of managing patients with underlying health conditions during a possible upcoming new pandemic. This information can be used to guide the development of clinical guidelines and protocols for managing patients with newly emerged coronaviruses in the future, as well as to inform the development of therapeutic approaches and vaccination strategies. Overall, this review article on COVID-19 in patients with anemia and hematological malignancies is an important tool in preparing for and managing future outbreaks of newly emerged coronaviruses.

## Data Availability

Not applicable.

## Abbreviations

ACS: Acute chest syndrome
ALL: Acute lymphoid leukemia
AML: Acute myeloid leukemia
AI: Anemia of inflammation
APS: Antiphospholipid syndrome
AA: Aplastic anemia
AIHA: Autoimmune hemolytic anemia
CNS: Central nervous system
CAR: Chimeric antigen receptor
CLL: Chronic lymphocytic leukemia
CML: Chronic myeloid leukemia
CSF: Colony-stimulating factor
COVID-19: Coronavirus disease 2019
CRS: Cytokine release syndrome
CS: Cytokine storm
DBA: Diamond-blackfan anemia
DIC: Disseminated intravascular coagulation
EBV: Epstein-Barr virus
ET: Essential thrombocytopenia
FLT3-ITD: FMS-like tyrosine kinase-3 internal tandem duplication
GI: Gastrointestinal
HDMs: Haematological disorders and malignancies
Hb: Hemoglobin
HLH: Hemophagocytic lymphocytosis
HS: Hereditary spherocytosis
HL: Hodgkin’s lymphoma
hATG: Horse antithymocyte globulin
HIV: Human immunodeficiency virus
HIF-1α: Hypoxia-inducible factor 1 alpha
IFN-γ: Interferon gamma
IL-1β: Interleukin-1 beta
IgG: Immunoglobulin G
ITP: Immune thrombocytopenic purpura
IL: Interleukin
KD: Kawasaki disease
LER: Leukoerythroblastic reaction
MAS: Macrophage activation syndrome
MCL: Mantle cell lymphoma
MAbs: Monoclonal antibodies
MM: Multiple myeloma
MOF: Multiple-organ failure
MPN: Myeloproliferative neoplasm
NK: Natural killer
NHL: Non-hodgkin’s lymphoma
ORF: Open reading frames
OS: Oxidative stress
PV: Polycythemia vera
PPIX: Protoporphyrin IX
ROS: Reactive oxygen species
RBCs: Red blood cells
SARS-CoV-2: Severe acute respiratory syndrome coronavirus 2
SCD: Sickle cell disease
SLL: Small lymphocytic lymphoma
SDIs: Sociodemographic indices
TNF-α: Tumor necrosis factor alpha
TKIs: Tyrosine kinase inhibitors
